# Factors Promoting Development of Fibrosis in Crohn’s Disease

**DOI:** 10.3389/fmed.2017.00096

**Published:** 2017-07-07

**Authors:** Gerhard Rogler, Martin Hausmann

**Affiliations:** ^1^Department of Gastroenterology and Hepatology, University Hospital, University of Zurich, Zurich, Switzerland; ^2^Zurich Center for Integrative Human Physiology (ZIHP), University of Zurich, Zurich, Switzerland

**Keywords:** fibrosis, Crohn’s disease, animal model, transforming growth factor β, collagen, stricture, therapy

## Abstract

The concepts on the pathophysiology of intestinal fibrosis in Crohn’s disease (CD) have changed in recent years. Some years ago fibrosis was regarded to be a consequence of long-standing inflammation with subsequent destruction of the gut wall matrix followed by scar formation and collagen deposition. Fibrosis in CD patients appeared to be an irreversible process that could hardly be influenced. Therefore, the main target in CD therapy was to control inflammation to avoid fibrosis development. Many of these assumptions seem to be only partially true. Inflammation may be a necessary prerequisite for the initiation of fibrosis. However, when the pathophysiologic processes that lead to fibrosis in CD patients have been initiated fibrosis development may be independent of inflammation and may continue even when inflammation is under good medical control. Fibrosis in CD also may be reversible. After strictureplasty local collagen deposits decrease or even disappear. With new animal models for intestinal fibrosis on the horizon, we need to spend more efforts on understanding the factors influencing fibrosis in CD patients to finally find specific therapies. In this context, it will be as important to find markers and quantitative imaging tools to have reliable endpoints for clinical trials in fibrosing CD.

## Introduction

Fibrosis in general can be characterized as exaggerated accumulation of collagen-rich extracellular matrix (ECM) in a tissue normally containing much less connective tissue with permanent or transient local expansion of mesenchymal cells or mesenchymal like cells and subsequent impairment of organ function (1).

Traditionally, fibrosis in Crohn’s disease (CD) has been seen as a relatively slow process needing many months to develop (2). In the discussion of delayed diagnosis of CD, it is usually emphasized that stricturing complications of CD and severe fibrosis of CD intestine could by avoided by a timely diagnosis (3). However, recent data indicate that this may not necessarily be the case. A rapid development of fibrosis in some patients seems to be possible. Rapid reoccurrence of fibrosis has been described in patients that undergo liver transplantation for hepatic fibrosis or cirrhosis (4). Rapid lung fibrosis could be induced by inhalative toxins in animal models (5, 6), and rapid liver fibrosis is seen in some models of primary sclerosing cholangitis (7). These data and further evidence support the concept that under certain circumstances fibrosis and subsequent stricture formation in some CD patients may be much faster than traditionally assumed.

It is evident from clinical findings that fibrosis only develops in segments of the gut where inflammation in the context of CD is present (1). Fibrosis in gut segments that never showed inflammatory involvement has not been reported. While this seems to be obvious, it is less clear what factors really trigger the process.

Another “dogma” also has been revised recently. It is no longer believed that only primary mesenchymal cells such as fibroblasts or smooth muscle cells can contribute to fibrosis in CD (1). Cells that contribute to fibrosis in CD patients may also derive from intestinal epithelial cells *via* a process called epithelial-to-mesenchymal transition (EMT) (1, 2, 8, 9) or from endothelial cells *via* endothelial-to-mesenchymal transition (EndoMT) (10).

A third important new aspect in the discussion is the assumption that fibrosis in CD may not be irreversible (11, 12). After strictureplasty in patients with CD suffering from clinical strictures the fibrosis in the gut wall was later on found to be reduced or even completely absent (13). Reversibility of fibrosis had been demonstrated before in other fibrotic diseases such as liver fibrosis (14).

Intestinal fibrosis subsequently is neither necessarily a very slow process nor completely dependent on the presence of inflammation, nor irreversible (1, 2, 9, 12, 15). Therefore, it appears to be important to review the cellular and molecular factors that contribute to fibrogenesis in CD.

## Factors Activating Matrix-Producing Cells

Matrix-producing cells are activated by paracrine signals, autocrine factors, and pathogen-associated molecular patterns derived from microorganisms or damage-associated molecular patterns that interact with pattern recognition receptors (1, 2, 12, 15). Transforming growth factor β (TGF-β) is an important mediator of mesenchymal cell activation. Its important role as a central regulator of fibrosis has been emphasized for many tissues and diseases (16–24). TGF-β expression is found to be upregulated in inflamed mucosa of inflammatory bowel disease patients (25–28). In addition, also inhibitory molecules of TGF-β action such as SMAD7 are upregulated in CD mucosa (29, 30). Recent therapeutic approaches now target SMAD7 expression by an antisense oligonucleotide (Mongersen) to allow more TGF-β action mainly of regulatory T-cells (31). It will be interesting to see whether a parallel activation of mesenchymal cells can be prevented (32). Data on mesenchymal cell activation and collagen deposition derived from clinical trials that are under way with Mongersen will help us to understand which role TGF-β plays for the activation of mesenchymal cells, for the initiation of EMT or EndoMT and for gut wall fibrosis in CD patients.

Other factors that play an important role in activating mesenchymal cells are activins (33), connective tissue growth factor (34–36), platelet-derived growth factor, insulin-like growth factor (IGF-1, -2), epidermal growth factor, and endothelins (ET-1, -2, -3) (2, 12, 15). All of those factors increase collagen synthesis by mesenchymal cells upon stimulation (2, 12, 15). The relative contribution of the respective factors and whether synergies are developed is unclear. Therefore, it is also unclear whether targeting one of those factors in an anti-fibrotic therapeutic approach would make sense (1).

Besides those specific factors inflammation *per se* is a strong activator of mesenchymal cells and also contributes to EMT and EndoMD (1, 8). Therefore, it has been assumed by many authors that control of inflammation would prevent the development of gut wall fibrosis. This seems to be questionable now. Recent epidemiological data indicate that biologicals have reduced the number of surgeries performed due to insufficient control of inflammation. However, despite effective and much better control of inflammation the development of CD in general from a B1 phenotype (only inflammatory) to a B2 (fibrotic) or B3 (penetrating) phenotype seems not to be significantly reduced (37, 38). This raises the important questions whether inflammatory mediators and molecules trigger the fibrotic process early and whether this process finally becomes independent from inflammation. If this would be the case—and there is quite some evidence to support this assumption—the development of anti-fibrotic therapies would be absolutely mandatory. If we cannot interfere with the progression of fibrosis in a significant number of patients with our current therapeutic armamentarium, the need for new drug development becomes obvious.

## Animal Models to Study Fibrosis-Promoting Factors and Potential Therapies

Several animal models for the study of intestinal fibrosis have been proposed and described (1). All of them have some advantages as well as disadvantages and none of them really resembles intestinal fibrosis of CD patients. Spontaneous intestinal fibrosis does not occur in rodent models, and therefore all models require some manipulation and artificial conditions.

The first models used to study intestinal fibrosis were models in which colonic inflammation was chemically induced, such as the trinitrobenzene-sulfonic acid (TNBS) and chronic dextran sodium sulfate (DSS) colitis in mice (1). Some collagen deposition and fibrosis is observed in these models. However, fibrosis is usually inconsistent, and the experimental duration until the occurrence of fibrosis limits the applicability of the mentioned mouse models. In addition, the contribution of the chemical trigger of the inflammation (TNBS or DSS) raises some concerns with respect to pathophysiological relevance. Similar to those chemically triggered models, the injection of the bacterial wall-derived compound peptidoglycan–polysaccharide into the gut wall induces inflammation and fibrosis (39). While this model is an example for fibrosis triggered by microbial products, it is unclear whether bacteria play an essential role in CD fibrosis. The SAMP1/Yit mouse was reported to develop spontaneous inflammation with ileitis and fibrosis (40, 41). However, the access to this model is limited, and the extent of fibrosis seems to depend on the vivarium the mice are bred in.

**Figure 1:**
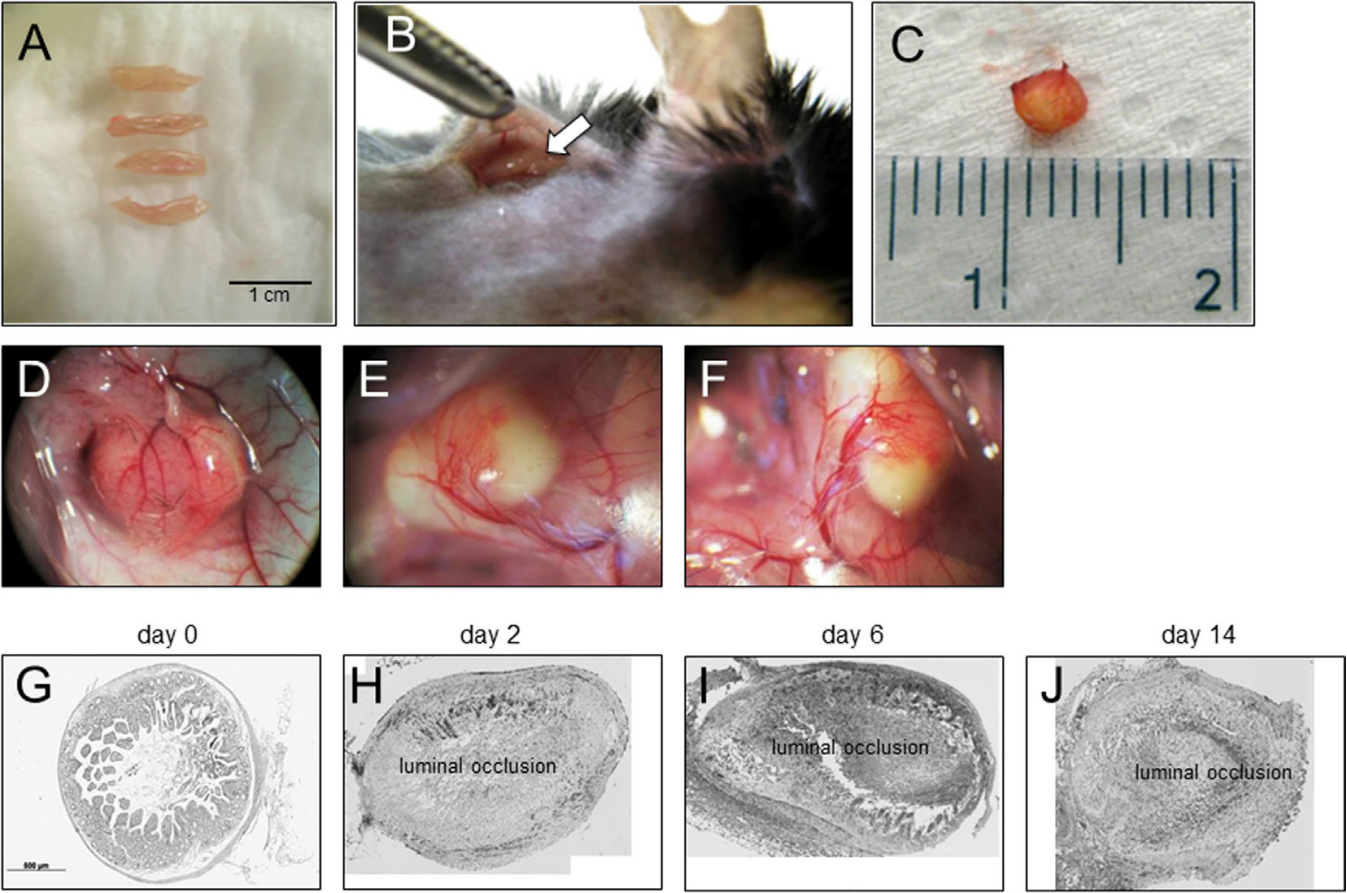
Heterotopic transplantation, revascularization, and luminal occlusion of the graft. **(A)** Small bowel resections are extracted from C57BL/6 mice. **(B)** For isogeneic transplantation, the resection (arrow) is implanted into subcutaneous tissue in the neck of C57BL/6 mice. **(C)** The graft is freed from the pouch and harvested from the neck of the recipient 14 days posttransplantation. **(D–F)** Grafts in the neck of recipient animals observed *in situ* present a decreased length but are otherwise macroscopically intact. Blood vessels from the surrounding tissue stretch toward the graft where they form a dense network (twofold magnification). **(G)** Histologic cross sections of freshly isolated small intestine (day 0). Small bowel resections are extracted from C57BL/6 mice, implanted into C57BL/6 mice for isogeneic transplantation, and explanted at **(H)** day 2, **(I)** day 6, and **(J)** day 14 after transplantation. Transmitted light microscopy, H&E staining. Grafts revealed luminal occlusion.

**Figure 2:**
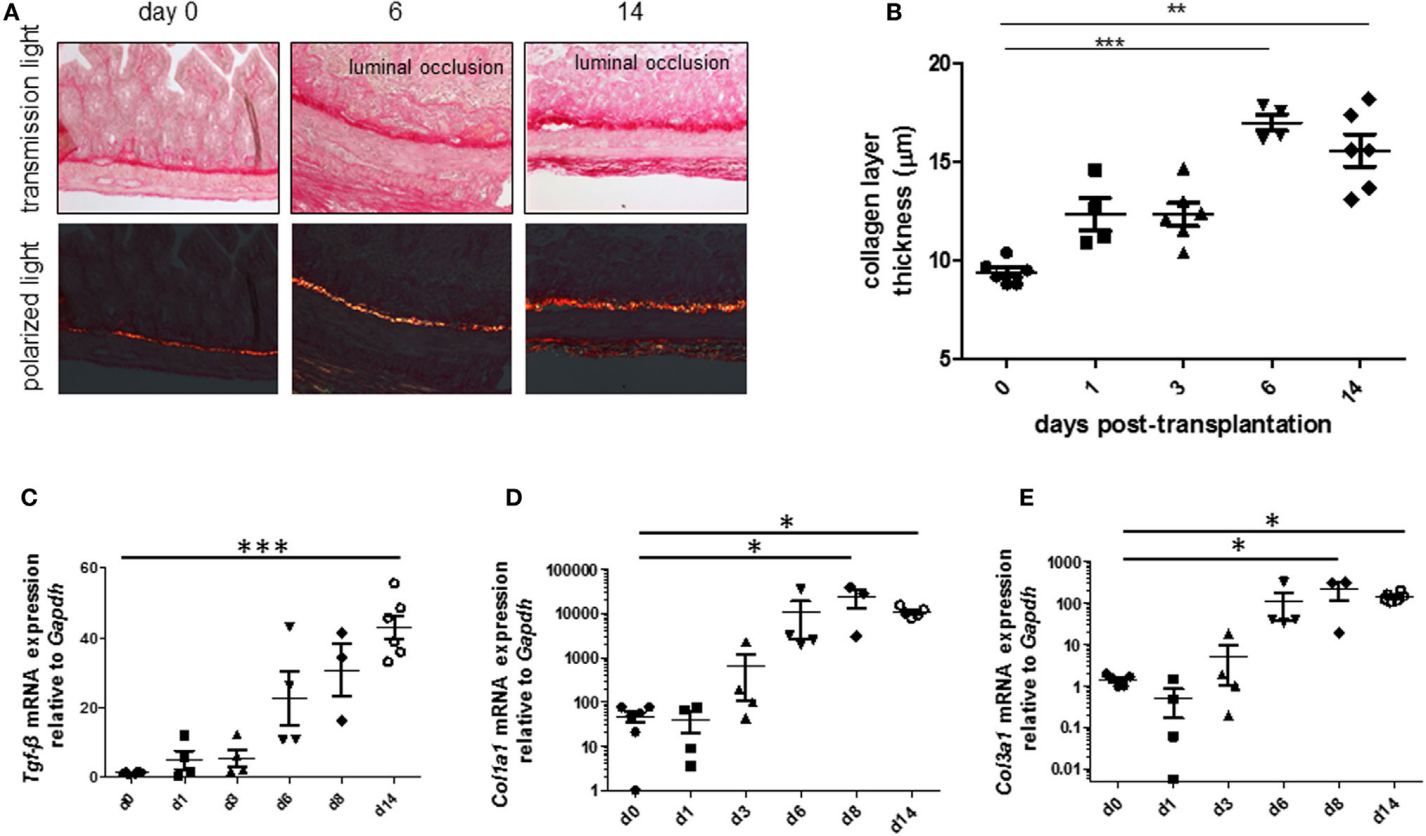
Collagen layer thickness and *Tgf-β, Col1a1*, and *Col3a1* mRNA are significantly increased in grafts from the heterotopic transplantation model in a time-dependent manner. Small bowel resections are extracted from RAG2 knockout mice and implanted into RAG2 knockout mice for isogeneic heterotopic transplantations. **(A)** Sirius Red staining. Transmission light microscopy and polarized light microscopy. **(B)** Collagen layer thickness measurement using transmission light microscopy confirmed significantly increased collagen layer thickness in a time-dependent manner (**p < 0.01, ***p < 0.001, ANOVA, Dunn’s multiple comparison test). Thickness was calculated from at least eight places in representative areas at 10-fold magnification for each single graft. **(C)** *Tgf-β* qPCR. **(D)** *Col1a1*. **(E)** *Col1a3*. *p < 0.05, ***p < 0.001, Kruskal–Wallis test, Dunn’s multiple comparison test.

To be able to study therapeutic interventions with the target of inhibiting intestinal fibrosis we established a new—but still very artificial model. For the study of bronchiolitis obliterans and bronchial fibrosis, pulmonologists had developed a heterotopic transplant model of trachea in rats (42). We adopted this model and investigated whether the heterotopic transplantation of small intestine into the neck fold of rats would also be followed by the development of fibrosis. Indeed, we detected a rapid fibrosis of the small intestinal wall occurring within 2 weeks (43). This was associated with increased expression of typical mediators of fibrosis such as αvβ6 integrin, IL-13, and TGF-β (43). Further, we detected a loss of intestinal epithelium morphology, could demonstrate exaggerated collagen deposition, which led to luminal wall thickening culminating in a veritable fibrotic occlusion of the intestinal lumen (43). As the available reagents to study fibrosis in rats are limited and it was desirably to study certain knockout or transgenic animal models we investigated whether the heterotopic transplant model also would work in mice (Figures 1A–C). As expected, we found a similar time course of development of fibrosis in the mouse model (44). C57BL/6 mice are used as donors for isogeneic transplantation into C57BL/6 recipients in this model. Interestingly, a rapid revascularization occurs in the intestinal grafts in the neck fold (Figures 1D–F). In small intestinal grafts isolated up to 21 days after transplantation, the lumen was obstructed by granulation tissue and fibrotic material (Figures 1G–J and (44)). The grafts partially had lost their typical crypt structure which in some specimen occurred already at day 2 after transplantation indicating that hypoxia may have an important role for this development. Collagen layer thickness was observed to be significantly increased in grafts in a time-dependent manner [Figures 2A,B; (44)]. Confirmatively, *Tgf-β* and collagen mRNA was observed to be significantly increased in a time-dependent manner [Figures 2C–E; (44)].

We used this newly developed model to study established anti-fibrotic drugs and their effect on the development of fibrosis (44). Pirfenidone so far is the best established therapy for idiopathic lung fibrosis (45–47). When we applied pirfenidone three times a day for 6 days by oral gavage, we found that the collagen layer was significantly decreased in comparison to the collagen layer thickness in grafts from vehicle treated mice (44). Similar, TGF-β mRNA expression was significantly decreased upon pirfenidone treatment compared to vehicle (44).

## Factors Involved in Tissue Remodeling

Additional factors involved in intestinal fibrosis that have not been discussed so far regulate the turnover of the ECM (2, 12, 15, 48). It is generally assumed that in normal tissue, i.e., in the normal intestinal wall there is a fine balance between ECM production and degradation (1). This balance is maintained on one hand by matrix metalloproteinases (MMPs) that break down and degrade ECM, and on the other hand tissue inhibitors of matrix metalloproteinases (TIMPs) that counteract this degrading activity. Under pathophysiologic conditions, when ECM production is increased and surpasses degradation intestinal fibrosis will occur. In human, CD strictures increased expression of MMPs, and also TIMPs has been observed. However, it is difficult of course to functionally investigate the balance and dynamics between the different pro-degrading and degradation-inhibiting proteins and mechanisms.

Further functions of MMP-9 include the regulation of cell migration, invasion, cell signaling as well as induction and regulation of EMT in multiple tissues (49–51). In fact, MMP-9 is the most abundantly expressed tissue degrading and remodeling protease in inflamed CD tissue (52). In biopsies from CD patients, MMP-9 was found as latent (pro-) and mature form (53). Further, serum and urinary levels of MMP-9 correlate with disease activity in CD patients. It has been suggested that MMP-9 serum levels could be a useful marker of CD disease activity in children. In DSS colitis in mice, targeted deletion of MMP-9 has a protective effect, whereas mice overexpressing MMP-9 develop more severe colitis (53).

As MMPs are obviously involved in intestinal fibrosis, we determined the expression of tissue remodeling proteases MMP-2, -9, -13, and TIMP-1 in our heterotopic transplant model by real-time PCR. When mice were treated with pirfenidone, a significant decrease in MMP-9 mRNA expression was observed (44). Similar, MMP-2, -13, and TIMP-1 mRNA expression was decreased upon pirfenidone (44).

Further, we investigated, whether the therapeutic neutralization of MMP-9 by specific antibodies would alter the development of fibrosis in the heterotopic transplant model. When we treated mice in our model with two different anti-MMP-9 antibodies, the lumen of the intestinal grafts was only partially obstructed, and some crypt structures were still present (53). Whereas the collagen layer was much thicker in grafts harvested from the isotype control-treated group, grafts harvested from anti-MMP-9 antibody-treated mice showed almost “normal” collagen layer thickness (53). Treatment with the two anti-MMP-9 antibodies was followed by lower accumulation of newly synthesized collagen, significantly thinner collagen layer, and lower collagen-specific amino acid hydroxyproline. Expression of MMP-9 and TIMP-1 were not significantly changed by the MMP-9 antibody treatment (53). When we assessed gelatinase activity in homogenates from our grafts by zymography and by ELISA, all day-14 explants exhibited increased total MMP-9, and the MMP-9 antibody treatment was followed by some reduction of MMP-9 activity in the explants (53).

## Summary

We have just started to more specifically understand the factors and pathways that lead to intestinal fibrosis. This is necessary to address the high clinical need of focused treatment of fibrosis in CD patients. New animal models may be helpful to screen for successful therapies. In some models, such as the heterotopic transplant model of small intestinal segments, pirfenidone and anti-MMP-9 antibodies have provided promising results. Further studies will be necessary to confirm these results and to find additional factors promoting development of fibrosis in CD.

## Author Contributions

GR: writing of the manuscript. MH: critical revision of the manuscript and compilation of the figures.

## Conflict of Interest Statement

GR discloses grant support from AbbVie, Ardeypharm, MSD, FALK, Flamentera, Novartis, Roche, Tillots, UCB, and Zeller. MH discloses grant support from AbbVie and Novartis.
